# Unique genomic and neoepitope landscapes across tumors: a study across time, tissues, and space within a single lynch syndrome patient

**DOI:** 10.1038/s41598-020-68939-7

**Published:** 2020-07-22

**Authors:** Tanya N. Phung, Elizabeth Lenkiewicz, Smriti Malasi, Amit Sharma, Karen S. Anderson, Melissa A. Wilson, Barbara A. Pockaj, Michael T. Barrett

**Affiliations:** 1 School of Life Sciences, Arizona State University, Tempe, AZ 85282 USA; 2 Center for Evolution and Medicine, Arizona State University, Tempe, AZ 85282 USA; 3 Division of Hematology-Oncology, Mayo Clinic in Arizona, Scottsdale, AZ 85259 USA; 4 The Biodesign Institute, Arizona State University, Tempe, AZ 85282 USA; 5 Division of General Surgery, Section of Surgical Oncology, Mayo Clinic in Arizona, Phoenix, AZ 85054 USA

**Keywords:** Cancer genomics, Genome informatics, Cancer

## Abstract

Lynch syndrome (LS) arises in patients with pathogenic germline variants in DNA mismatch repair genes. LS is the most common inherited cancer predisposition condition and confers an elevated lifetime risk of multiple cancers notably colorectal and endometrial carcinomas. A distinguishing feature of LS associated tumors is accumulation of variants targeting microsatellite repeats and the potential for high tumor specific neoepitope levels. Recurrent somatic variants targeting a small subset of genes have been identified in tumors with microsatellite instability. Notably these include frameshifts that can activate immune responses and provide vaccine targets to affect the lifetime cancer risk associated with LS. However the presence and persistence of targeted neoepitopes across multiple tumors in single LS patients has not been rigorously studied. Here we profiled the genomic landscapes of five distinct treatment naïve tumors, a papillary transitional cell renal cell carcinoma, a duodenal carcinoma, two metachronous colorectal carcinomas, and multi-regional sampling in a triple-negative breast tumor, arising in a LS patient over 10 years. Our analyses suggest each tumor evolves a unique complement of variants and that vaccines based on potential neoepitopes from one tissue may not be effective across all tumors that can arise during the lifetime of LS patients.

## Introduction

Lynch syndrome (LS) is the most common human cancer predisposition syndrome^1^. It arises as a result of defective DNA mismatch repair (MMR) with loss of the post-replicative proofreading and editing system that ensures genome integrity. As a result, patients with LS are predisposed to a spectrum of cancers, notably carcinomas of the colorectum and endometrium. LS is associated with pathogenic germline variants in one of the four key MMR genes, mutL homologue 1 (*MLH1*), mutS homologue 2 (*MSH2*), mutS homologue 6 (*MSH6*) or postmeiotic segregation increased 2 (*PMS2*). Tumors arising in LS patients typically undergo loss of heterozygosity (LOH) of the wild type allele of the gene of interest resulting in complete loss of function of the protein. The genomic instability associated with MMR targets microsatellite repeats resulting in an accumulation of frameshift and insertion/deletion (indels) variants^2,3^. This microsatellite instability (MSI) signature is associated with responses to immune checkpoint inhibition (ICI) likely as a result of variant derived neoepitopes that may trigger the host immune system^4,5^. In contrast to other forms of genomic instability, notably associated with loss of BRCA function, MSI + tumors tend to be diploid with fewer chromosomal aberrations in their genomes^6–8^.

Clinical testing for LS includes immunohistochemistry (IHC) assays for expression of the four primary MMR genes and a panel of PCR markers for the presence of shifts in the number of dinucleotide and trinucleotide microsatellite repeats at selected loci^9^. The absence of expression for one or more of the 4 MMR genes and the presence of MSI defined by shifts in at least 2 of 5 microsatellite loci provides molecular confirmation for a diagnosis of LS. Despite a common molecular pathogenic basis the responses of MSI + tumors to ICI varies suggesting that either distinct variants or a threshold for elevated mutational burden trigger more pronounced immune responses^10^. Studies of mutational burdens and neoepitopes have primarily focused on colorectal cancer (CRC), the most common tumor associated with MSI + status including LS. Reports have suggested that MSI + CRCs evolve a set of recurring somatic variants targeting both coding and noncoding loci that may be exploited for vaccine development^11^. However, unlike patients with sporadic MSI + tumors LS patients have a lifetime risk of multiple different types of cancers. Despite this, little is known about the mutational landscape and neoepitope profiles of tumors arising at multiple sites over time in individual LS patients.

**Figure 1:**
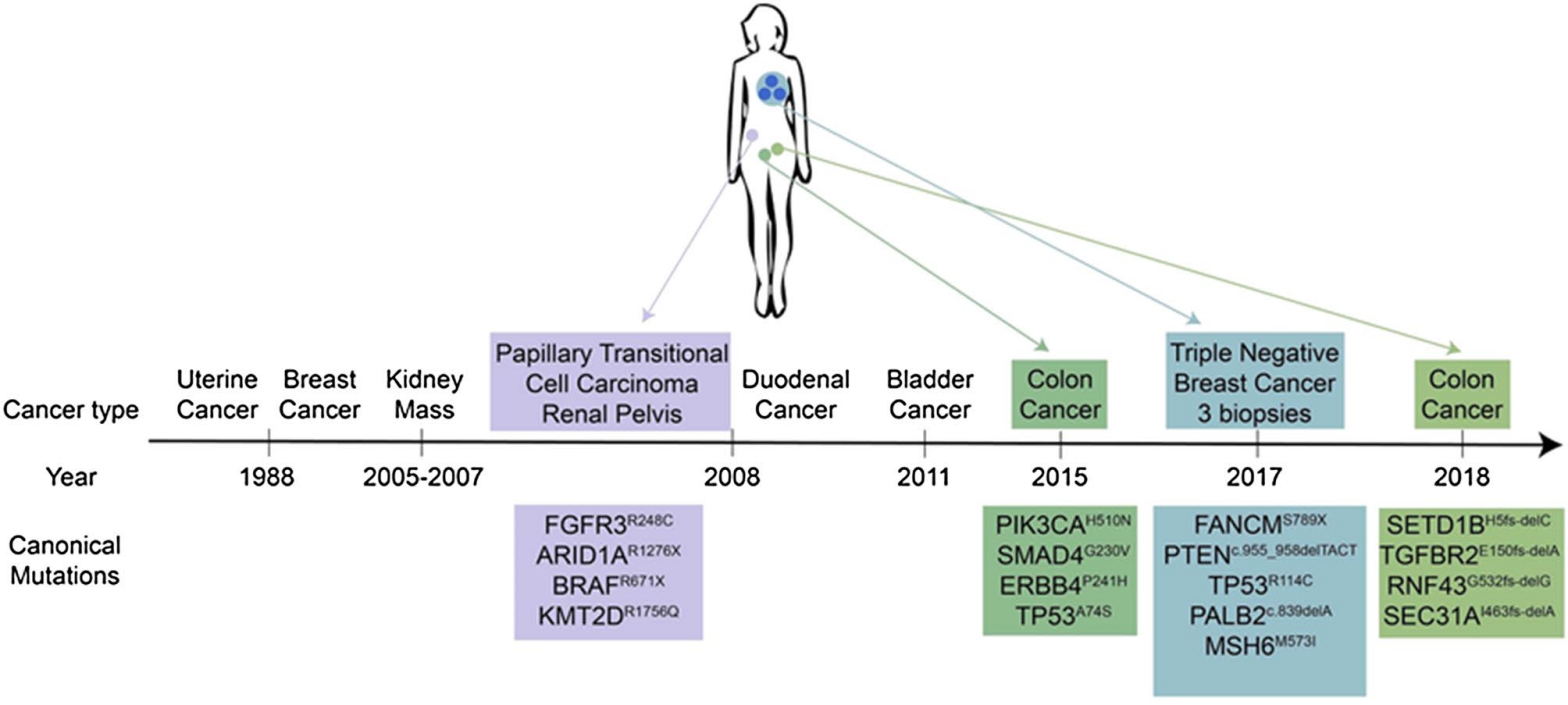
Longitudinal lynch syndrome patient tumor samples. Timeline and tissue specific variants for individual tumors arising in LS patient.

To investigate the longitudinal mutational patterns arising in LS associated tumors we interrogated the genomes of five different cancers that arose over a period of 10 years in a patient who underwent resection in the absence of chemotherapy and radiation for each cancer. These included a papillary transitional cell carcinoma (PTCC) in the renal pelvis, a duodenal carcinoma (DC), two separate CRCs that arose 3 years apart, and multiple regions of a triple negative breast cancer (TNBC). In each case we flow sorted tumor fractions from archived formalin fixed paraffin embedded (FFPE) tissue and profiled the tumor genomes with whole genome copy number variant (CNV) arrays and whole exome sequencing. These data were then used to identify the pathogenic variant underlying the diagnosis of LS, and to compare and contrast the CNV, mutational and neoepitope patterns across these divergent tumors that arose over a 10 year period. These results provide a unique analysis of distinct MSI + tumors arising in a single LS patient (Fig. 1).

## Results

### Identification of pathogenic LS variant

**Figure 2:**
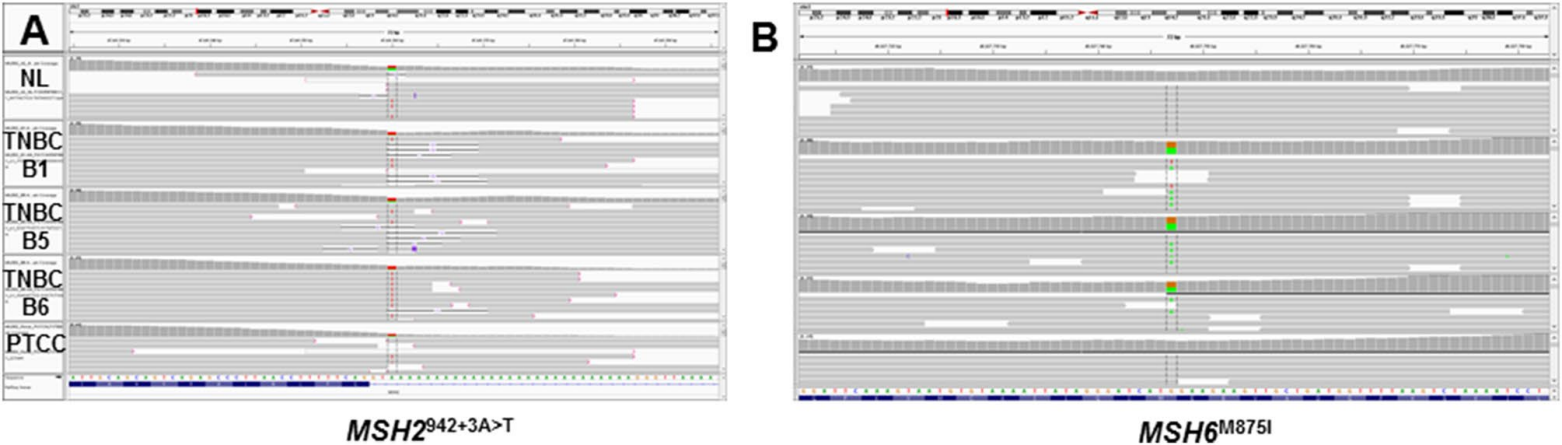
Variant analysis of MSH2 and MSH6. IGV views of: (**A**) heterozygous germ line and homozygous somatic *MSH2*^942+3A>T^ variant and (**B**) heterozygous somatic *MSH6*^M875I^ variant.

A common feature of tumors arising in LS patients is the loss of wild type alleles for the affected MMR gene. Although IHC and PCR testing in 2011 confirmed the diagnosis of LS the pathogenic MMR gene variant remained to be determined. We used the exome data from each tumor and a patient matched normal tissue to screen all known MMR genes for germline pathogenic variants that included LOH of the wild type allele in the tumors. Given previous IHC results we focused on variants in *MSH2* and *MSH6* as candidates. We identified the pathogenic variant NM_000251.2 (MSH2):c.942 + 3A > T that was heterozygous in the germ line but had converted to homozygosity in each tumor profiled (Fig. 2). This is a common pathogenic *MSH2* variant associated with LS^12^. In contrast, none of the variants detected in MMR genes had allele patterns consistent with a pathogenic role in the predisposition to cancer (Supplementary Table S1). Strikingly there was a somatic *MSH6*^M875I^ variant in the TNBC samples. The lack of MSH6 IHC staining is consistent with the presence of this likely pathogenic variant giving rise to a double negative MSI + tumor^13,14^. In contrast to *MSH2* this variant was heterozygous suggesting that additional events such as epigenetic silencing may have contributed to the lack of MSH6 expression. However there was an absence of MSH2 and MSH6 expression in both the TNBC from 2017 and the CRC from 2018, the latter lacking the somatic *MSH6* variant. Notably lack of MSH6 staining is known to occur due to disruption of MSH2-MSH6 heterodimer complexes and the degradation of MSH6 in the presence of a homozygous pathogenic *MSH2* variant^15,16^.

### Distinct driver mutations, CNV profiles and ploidies in each LS cancer

**Figure 3:**
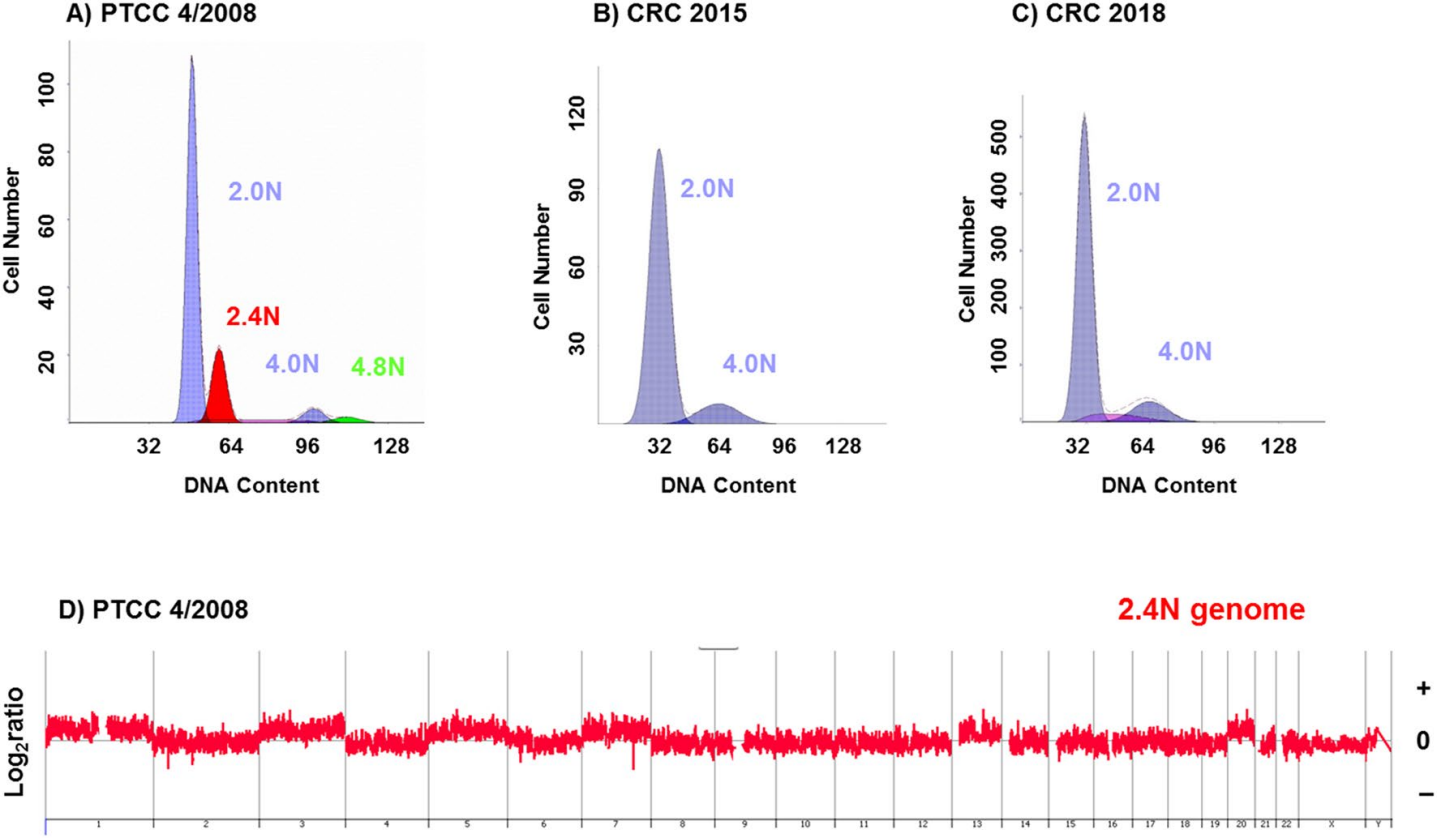
Flow cytometry and CNV profiles of PTCC, duodenal carcinoma, and CRCs in LS patient. (**A**–**D**) DNA content histograms of flow sorted tumor biopsies of near diploid (2.4N) PTCC and diploid (2.0N) duodenal carcinoma and CRCs. (**E**,**F**) CNV profiles of flow sorted 2.4N PTCC and 2.0N duodenal carcinoma tumor fractions. The X and Y axes in the CGH plots represent chromosome and log_2_ratios.

**Figure 4:**
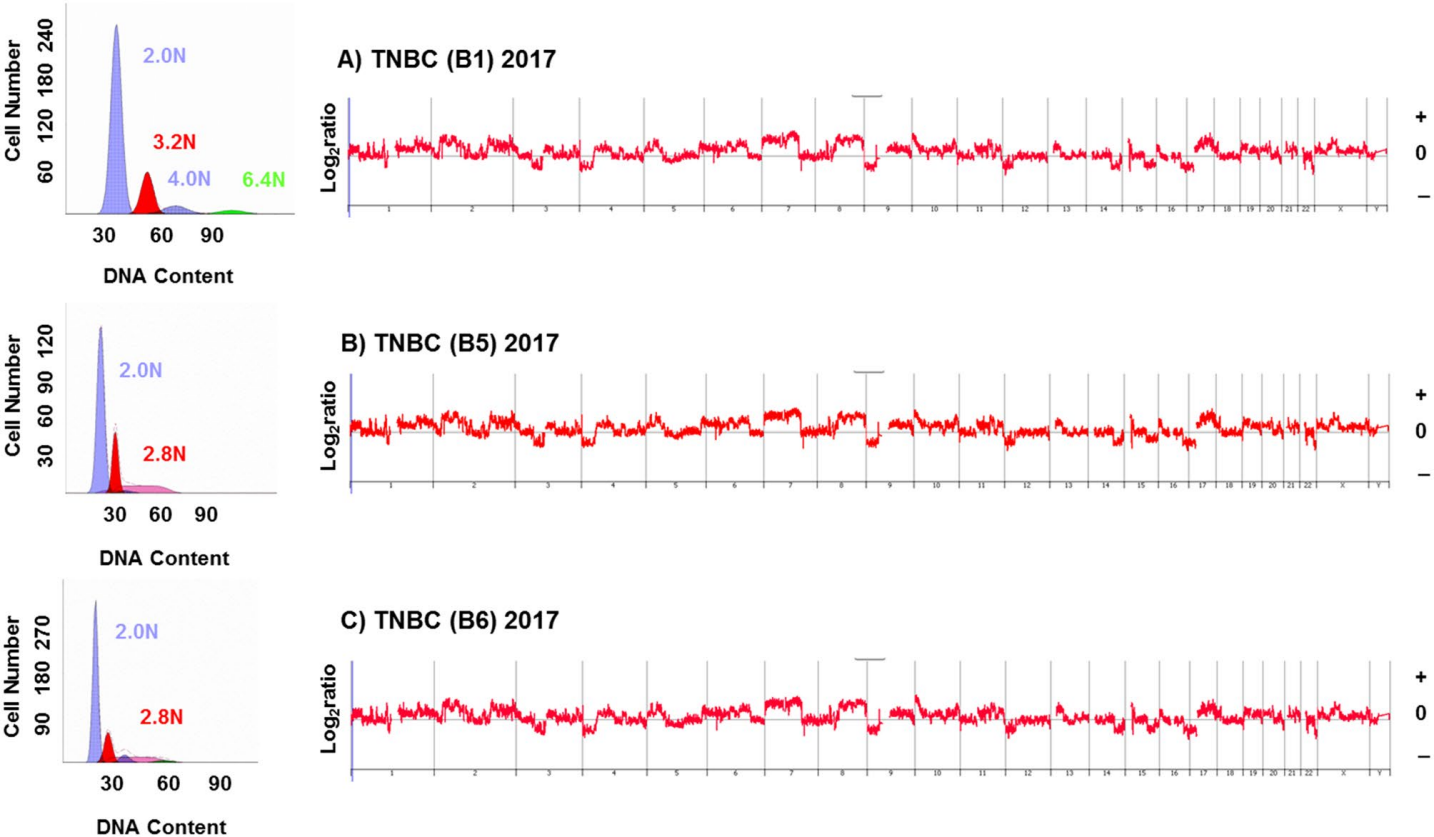
Flow cytometry and CNV profiles of biopsies from TNBC in LS patient. (**A**–**C**) DNA content histograms (left panels) identify distinct aneuploid populations in each biopsy. The CNV profiles are matched in each sorted population (right panels). The X and Y axes in the CGH plots represent chromosome and log_2_ratios.

Tumor genomes that were diploid, duodenal carcinoma (2008) and the two CRCs (2015, 2018), or near diploid, PTCC (2008) by DNA content contained low levels of CNVs (Fig. 3). The absence of chromosomal instability is frequently seen in MSI + tumors^6,8^. In contrast three biopsies from different regions within the TNBC (2017) had 2.8 N and 3.2 N ploidies (Fig. 4). In addition the genomes of each of the three sorted TNBC fractions had high levels of CNVs that included every chromosome consistent with a BRCA like phenotype. Despite this additional chromosomal instability and variation in ploidies the CNV patterns were identical across the three sorted populations.

A significant feature of this study is that all biopsies were from chemoradiation naive surgically resected tissues. Thus the pattern of variants and the predicted neoepitopes recapitulate the natural history of the tumors. Whole exome sequences were obtained from the PTCC, the two CRCs, and the TNBC sorted samples. Each tumor had unique variants that reflected the tissue of origin (Fig. 1, Table 1). For example the PTCC from 2008 had a *FGFR3*^R248C^ pathogenic variant that is enriched in upper urothelial tract cancers arising in LS patients^17^; the CRC from 2015 had variants of unknown significance (VUS) in *PIK3CA*^H510N^ and *SMAD4*^G230V^ whereas the CRC from 2018 had somatic indels in *TGFBR2* and *RNF43,* all of which are frequent somatic targets in CRCs^6^, while the TNBC from 2017 had a frame shift indel in *PTEN* and a *FANCM*^S789X^ nonsense variant in each of the three sorted biopsies. These variant patterns are consistent with the independent nature of each tumor.

**Table 1:** Tissue specific mutations.

PTCC (2008)	CRC (2015)	TNBCC (2017)	CRC (2018)
*FGFR3*^R248C^	*PIK3CA*^H510N^	*RANCM*^S789X^	*SETD1B*^H5fs-delC^
*ARID1A*^R1276X^	*SMAD4*^G230V^	*PTEN*^c.955_958delACTT^	*TGFBR2*^E150fs-delA^
*BRAF*^R671X^	*ERBB4*^P241H^	*TP53*^R114C^	*RNF43*^G532fs-delG^
*KMT2D*^R1756Q^	*TP53*^A74S^	*PALB2*^c.839delA^	*SEC31A*^I463fs-delA^
		*AIM2*^c.1026_1027delAA^	
		*MSH6*^M573l^	

### Heterogeneity in different cancer types from the same LS patient

**Figure 5:**
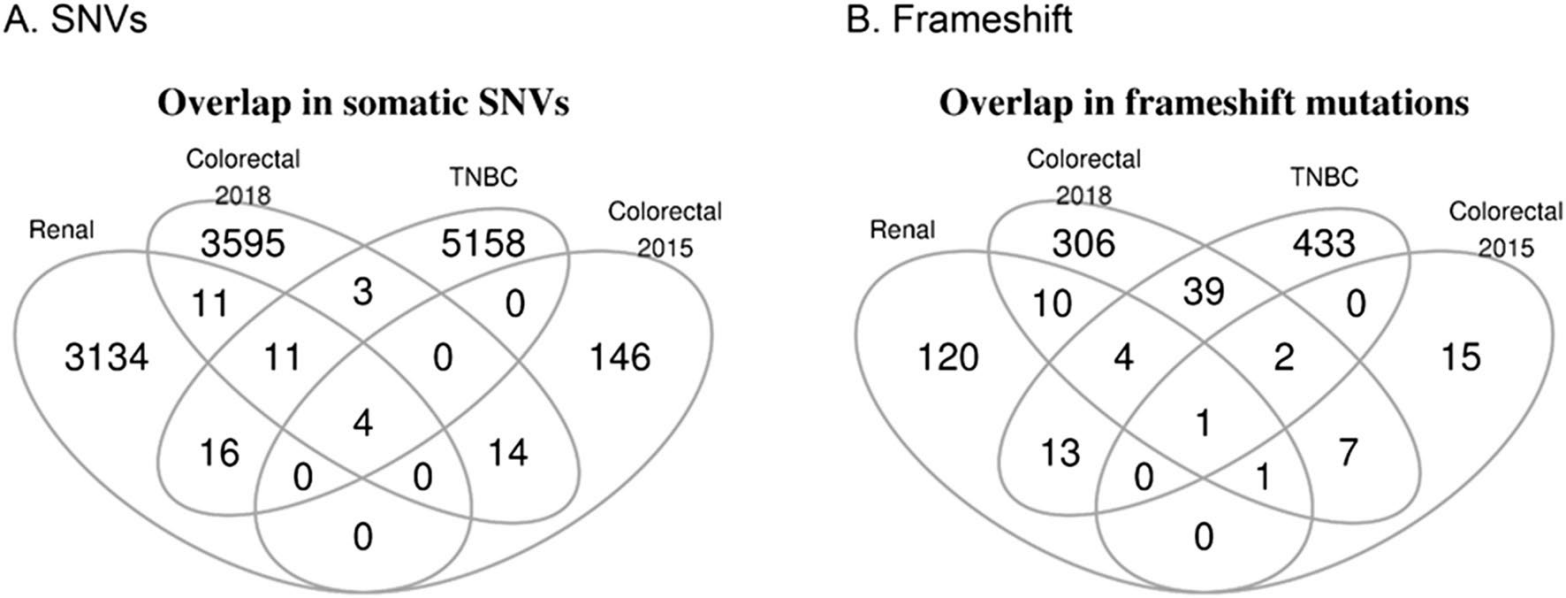
Overlap in somatic variants across 4 tumors in LS patient. Venn diagrams of: (**A**) Single nucleotide variants (SNVs) and (**B**) frameshift variants from whole exome data of PTCC (renal 2008), CRC (2015), TNBC (2017), and CRC (2018).

We observed limited overlap in somatic single nucleotide variants (SNVs) and frameshift variants (Fig. 5). These observations suggest that these cancers arise independently and the mutational landscape, with rare exceptions, differs across these cancers. Even though these four cancers arise from the same patient at different time points, it is highly likely that independent somatic variants give rise to each cancer, in the genetic background of LS. However we found four somatic likely benign SNVs that are shared across the four different cancers (Supplementary Tables S2, S3). These variants targeted four genes: *CROCCP2* (upstream gene variant), *PTPRD* (intronic variant), *ACOT2* (synonymous variant), and *RCAN1* (intronic variant). In addition we found one shared frameshift VUS targeting the epigenetic regulator *KMT2C* (Supplementary Fig. S1). The latter is frequently mutated in a variety of cancers^18,19^.

**Figure 6:**
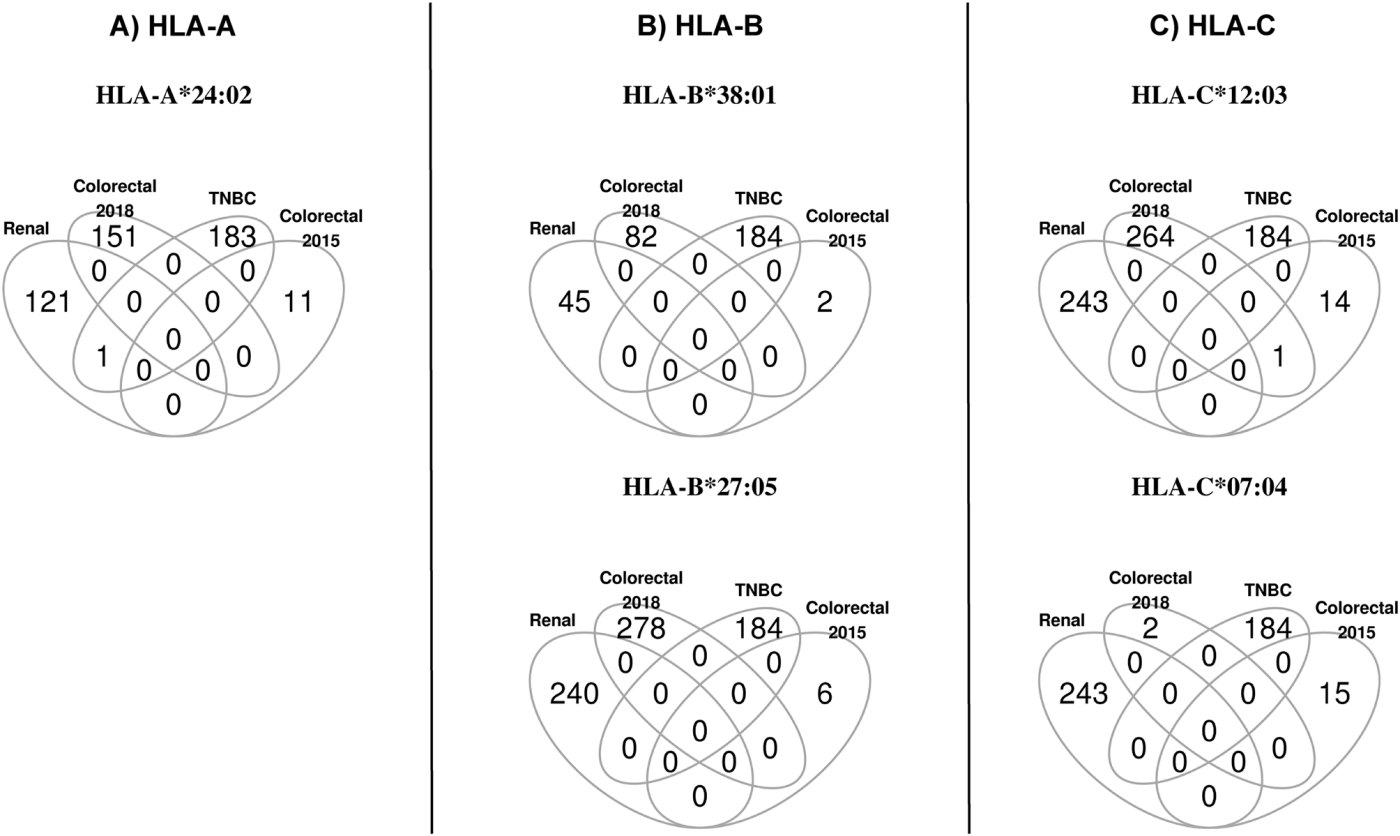
Overlap in predicted neoepitopes across 4 tumors in LS patient. Venn diagrams of predicted neoepitopes: (**A**) HLA-A, (**B**) HLA-B, and (**C**) HLA-C binding single nucleotide variants (SNVs) and frameshift variants from whole exome data of PTCC (renal 2008), CRC (2015), TNBC (2017), and CRC (2018).

Consistent with very little overlap in SNVs and frameshift variants across these cancers, we found no overlap in potential neoepitopes (Fig. 6). These results suggest that vaccines that are developed based on potential neoepitopes of one tissue may not work well across all tissues in LS patients.

### Multiregional sequencing and heterogeneity within the triple negative breast cancer from the LS patient

**Figure 7:**
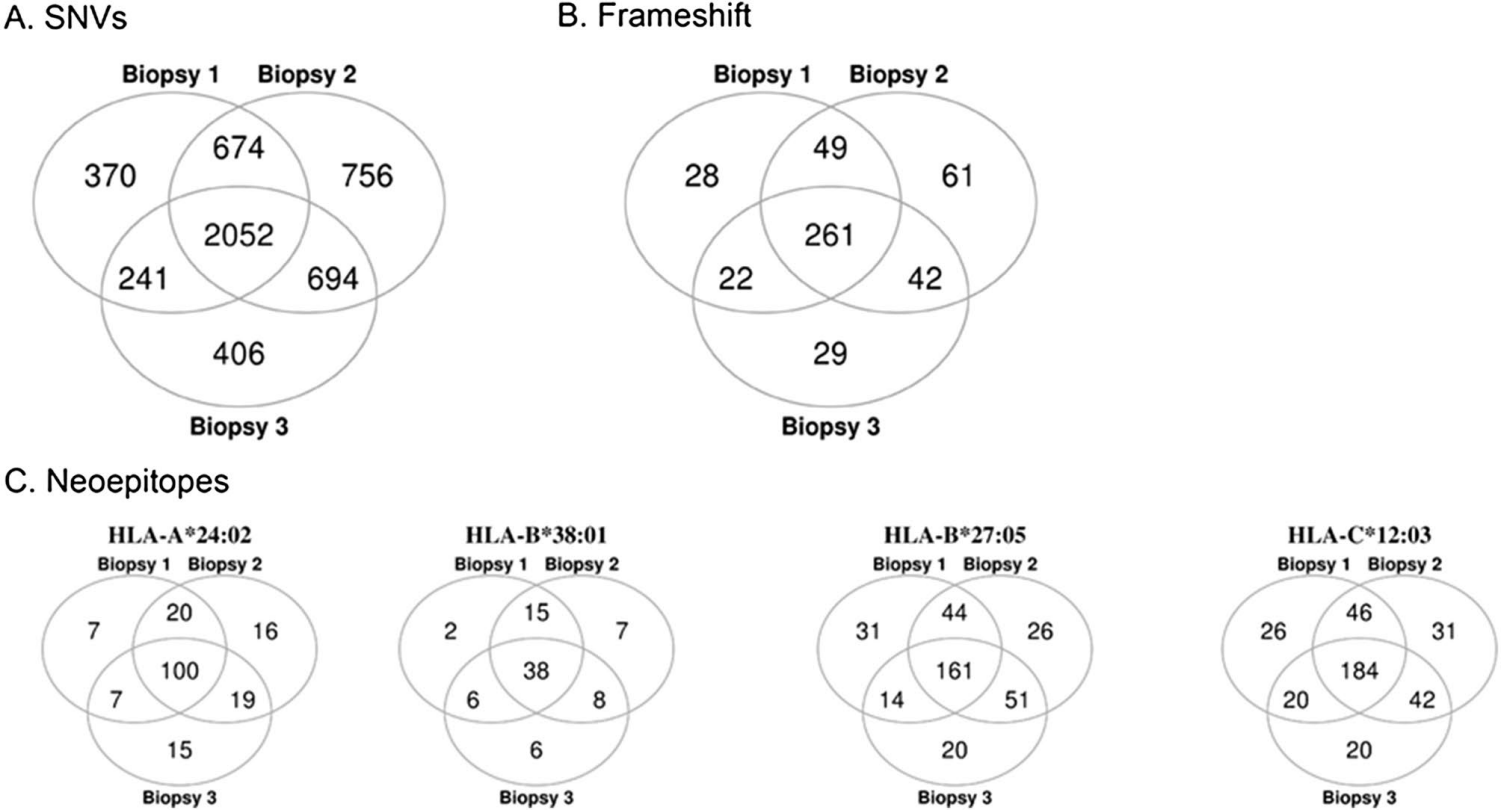
Overlap in variants and predicted neoepitopes across 3 biopsies from TNBC in LS patient. Venn diagrams showing overlap in three TNBC biopsies of: (**A**) SNVs, (**B**) frameshift variants, and (**C**) predicted neoepitopes.

We observed a large degree of heterogeneity across multiregional sequencing from three TNBC biopsies for the same breast tissue. Out of 5,193 total SNVs found across three biopsies, 2,052 (~ 40%) are shared (Fig. 7A). Out of 492 total frameshift variants found across three biopsies, 261 (~ 53%) are shared (Fig. 7B). The proportion of potential neoepitopes that are shared across three biopsies for HLA-A*24:02, HLA-B*38:01, HLA-B*27:05, and HLA-C*12:03 are 53%, 54%, 46%, and 50%, respectively (Fig. 7C). These observations suggest that this subset of neoepitopes could potentially work as therapeutics for targeting the TNBC.

### Similarities and differences between two metachronous LS colon cancers

**Figure 8:**
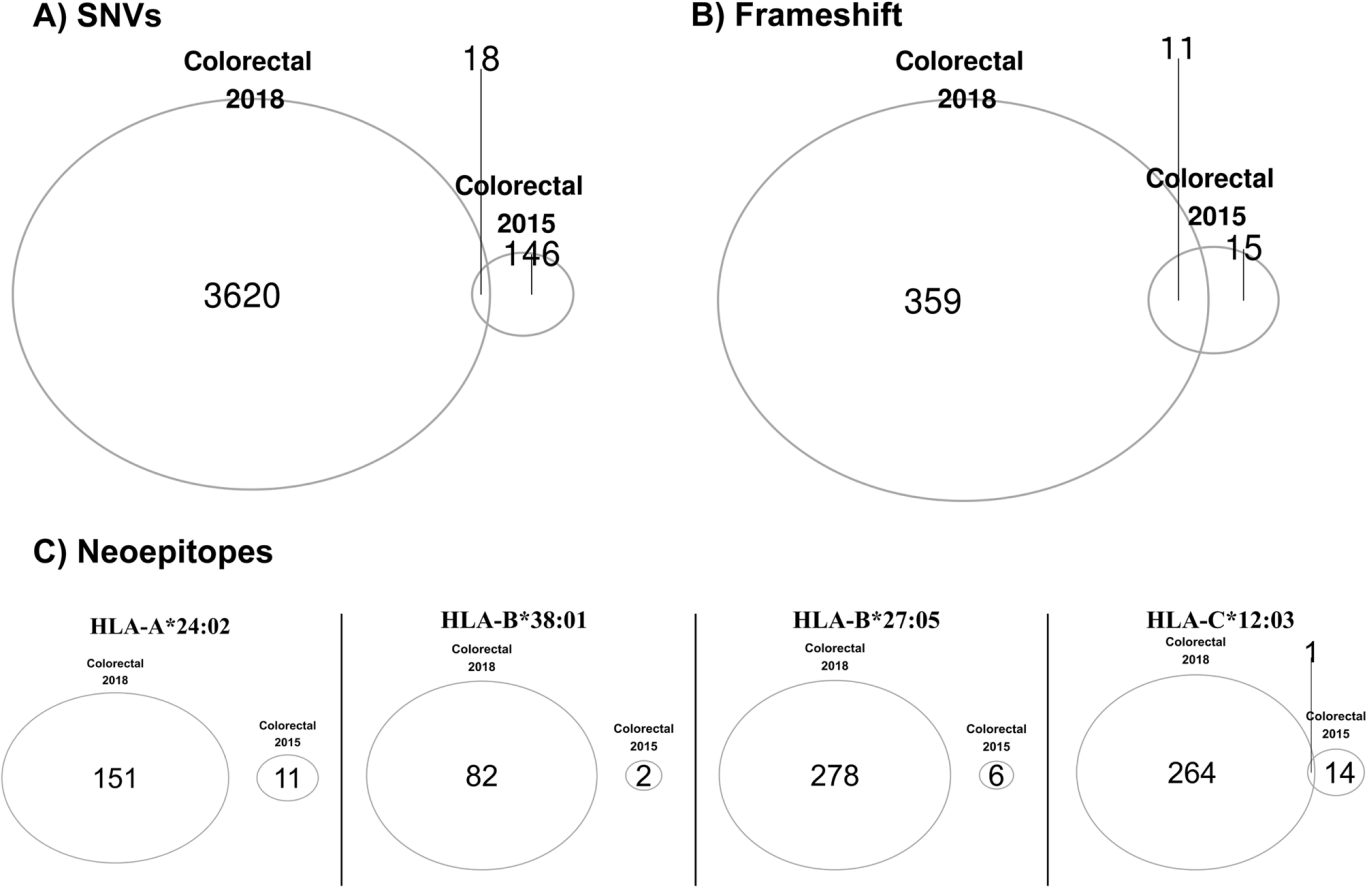
Overlap in variants and predicted neoepitopes in metachronous CRCs in LS patient. Venn diagrams showing overlap in: (**A**) SNVs, (**B**) frameshift variants, and (**C**) neoepitopes in the two colon cancers.

The initial CRC was resected in 2015 and was an adenocarcinoma with mucinous features that included 3/30 positive lymph nodes. The second CRC was resected in 2018 and was also a low grade adenocarcinoma with mucinous features but without any lymph node involvement. In contrast to the three breast biopsies sharing about 50% of SNVs, frameshift variants, and potential neoepitopes, we observed significantly less overlap between the two CRCs: only 0.5% of SNVs and 3% of frameshift variants are shared (Fig. 8A,B). Further, in only one of the HLA, HLA-C*12:03, we found one potential neoepitope that is shared between these two colorectal cancers (Fig. 8C). These results indicate that these two colorectal cancers are separate cancers and they arose independently of each other.

## Discussion

Our study provides a unique portrait of the genomic and neoepitope landscapes across four distinct chemoradiation naive tumors arising over a ten year period in a LS patient. These include a PTCC, two metachronous CRCs, and multi-regional analysis of a TNBC. In each case we flow sorted tumor nuclei from FFPE tissue to increase the resolution of our genomic analyses including whole genome CNV and whole exome profiles. Each tumor contained genomic features that are specific to the tissue of origin and the subtype of tumor. Notably the PTCC had a *FGFR3*^R248C^ pathogenic variant that is a recurring driver lesion for this tumor, while the TNBC had BRCA-like CNV features including high levels of interstitial aberrations and a somatic nonsense variant in *FANCM*. The latter has been identified as a breast cancer predisposition gene that confers an increased risk of TNBC^20,21^.

The tumor samples were sequenced at different times during our study. Furthermore FFPE tissues may harbor various artifacts that can interfere with genomic analyses. However we applied a rigorous variant calling pipeline with identical filters across all samples for variant calling. This included remapping all reads for each flow sorted tumor sample to the most recent 1,000 genomes HG38 build of the human genome. The PTCC and the multiple biopsies from the TNBC had aneuploid DNA contents that were exploited to flow sort tumor populations for genomic analyses. In contrast the duodenal and two metachronous CRCs were diploid by flow cytometry. In these cases we gated on the 4N(G_2_/M) fractions to enrich tumor nuclei for our analyses. There were variations in tumor purity in the later samples based on allele frequencies of somatic variants. Notably the 2015 CRC sample retained non-tumor nuclei in the sorted fractions that affected the exome results. Nevertheless we did not detect any shared somatic variants including those that were homozygous in the PTCC and TNBC sorted biopsies.

We examined the genes that contain at least one missense variant, the specific missense variations, and the potential neoepitopes that are shared across four different metachronous cancers arising over 10 years within the same LS patient (PTCC, two CRCs, and TNBC). We found little to no overlap in the genomes of these chemoradiation naive tumors, suggesting that tissue specific independent variants contribute to each of these cancers, instead of a common set of driver events. The lack of overlap in the predicted neoepitopes across these tumors suggests that a vaccine strategy for the lifetime risk of cancer in LS patients will be challenged by the diversity of each tumor including those arising in the same tissue. The one exception was a single base pair frame shift deletion at codon 2171 in *KMT2C*. Recurring frameshift deletions targeting this codon (COSV51277546) have been reported in various cancers^22^. Notably mutations and loss of expression of KMT2C and other members of this lysine methyltransferase family are associated with increased survival in pancreatic cancers^23,24^. Given the lengthy clinical history of this patient it will be of interest in future studies to explore the potential role of epigenetic dysregulation in LS patients.

## Methods

### Clinical samples

Tissue samples were obtained under a Mayo Clinic protocol 2130-00 Cancer Tissue Study (Principal Investigator Dr. B. A. Pockaj). This study was approved by Mayo Clinic IRB protocol 08-006579-08 Breast Cancer Clinical Genomics Project. The patient gave informed consent for the collection and use of the samples. All tumor samples were histopathologically evaluated prior to genomic analysis. All research conformed to the Helsinki Declaration (https://www.wma.net/policies-post/wma-declaration-of-helsinki-ethical-principles-for-medical-research-involving-human-subjects/). The LS patient’s oncologic history started in 1988 at age 53 with uterine cancer (Fig. 1). The patient underwent surgery with no additional therapy. She then started on hormonal replacement therapy and 9 months later developed a right breast palpable mass that was found to be cancer. She was treated with mastectomy and axillary lymph node dissection. Zero out of 20 lymph nodes was involved and she denied any tamoxifen therapy. She did well until 2005 when she was noted to have a kidney mass and until November 2007 when she had urinary tract infections and the same kidney mass that had not progressed. In 2008 she underwent a right nephroureterectomy for a grade 2, stage IA transitional cell carcinoma of the renal pelvis, with negative ureteral margins. In September of 2008 she was found to have a duodenal cancer and underwent a Whipple procedure which had a 10 × 8.5 cm infiltrating poorly differentiated carcinoma with medullary features of the duodenum. Four lymph nodes were removed and they were negative. The margins were also negative.

She was then followed and eventually was found to have bladder cancer in 2011. The patient’s tumor was tested for defective DNA mismatch repair in May 2011 using IHC for the four microsatellite DNA repair proteins (MSH2, MSH6, MLH1, PMS2) and PCR assays for five microsatellite loci (BAT25, BAT26, Mono27, NR24, and NR21). These clinical diagnostic tests and additional IHC analysis of MSH2 and MSH6 in TNBC (2017) and CRC (2018) tissues were done by the Department of Laboratory Medicine and Pathology, Mayo Clinic. All additional genomic profiling described in our manuscript was done in the setting of a research study. There was an absence of MSH2 and MSH6 staining as well as microsatellite instability (MSI) noted at 5 of 5 informative PCR markers confirming the MSI + nature of the tumor. In 2015 she was feeling poorly and workup found that she had a CRC. She underwent a right hemicolectomy which showed 3 colon cancers, one 6.5 cm in size, one 5.4 cm in size, the other 1.2 cm in size. These were low grade adenocarcinomas with partial mucinous features. The margins were negative. There was no lymphovascular invasion, however 3/30 lymph nodes were positive for metastatic disease. She was seen in consultation and declined any chemotherapy. In 2017 she presented with a contralateral or left breast cancer. Estrogen receptor (ER) and progesterone receptor (PR) were evaluated by standard ASCO/CAP guidelines with < 1% of the cells staining for the receptors respectively^25^. HER2 negative was defined by ASCO/CAP guidelines as staining by IHC of 0 or 1 + ^26^. HER2 IHC of 2 + was further evaluated by FISH and deemed negative by standard ASCO/CAP guidelines. Her most recent tumor in 2018 was an invasive mucinous CRC arising in a tubulovillus adenoma. The invasion involved submucosa (pT1) with negative (0/13) lymph nodes.

### Flow cytometry

Excess paraffin was removed from each FFPE sample with a scalpel from either side of 40–60 μm scrolls then processed according to our published methods^27,28^. We used one to three 50 µm scrolls from each FFPE tissue block to obtain sufficient numbers of intact nuclei for subsequent sorting and molecular assays. Nuclei from each sample were disaggregated then filtered through a 40 μm mesh prior to flow sorting with an Influx cytometer (Becton–Dickinson, San Jose, CA) with ultraviolet excitation and DAPI emission collected at > 450 nm. DNA content and cell cycle were analyzed using the software program MultiCycle (Phoenix Flow Systems, San Diego, CA).

### Copy number analysis

DNAs from flow sorted FFPE tissue were treated for one minute with DNAse 1 prior to Klenow-based labeling. In each case 1 ul of 10 × DNase 1 reaction buffer and 2 μl of DNase 1 dilution buffer were added to 7 μl of DNA sample and incubated at room temperature then transferred to 70 °C for 30 min to deactivate DNase 1. Sample and reference templates were then labeled with Cy-5 dUTP and Cy-3 dUTP respectively using a BioPrime labeling kit (Invitrogen, Carlsbad, CA) according to our published protocols^29^. All labeling reactions were assessed using a Nanodrop assay (Nanodrop, Wilmington, DE) prior to mixing and hybridization to CGH arrays (Agilent Technologies, Santa Clara, CA) for 40 h in a rotating 65 °C oven. All microarray slides were scanned using an Agilent 2565C DNA scanner and the images were analyzed with Agilent Feature Extraction version 11.0 using default settings. The aCGH data was assessed with a series of QC metrics then analyzed using an aberration detection algorithm (ADM2)^30^. The latter identifies all aberrant intervals in a given sample with consistently high or low log ratios based on the statistical score derived from the average normalized log ratios of all probes in the genomic interval multiplied by the square root of the number of these probes. This score represents the deviation of the average of the normalized log ratios from its expected value of zero and is proportional to the height h (absolute average log ratio) of the genomic interval, and to the square root of the number of probes in the interval.

### Sequence filtering, QC and alignment

DNAs from each sorted tumor population and a patient matched control sample were sequenced within the Mayo Clinic Medical Genome Facility (MGF) using established protocols for whole exome analysis. Briefly, whole exon capture was carried out with Agilent’s SureSelect Human All Exon 71 MB v6 kit. 500 ng of the prepped library is incubated with whole exon biotinylated RNA capture baits supplied in the kit for 24 h at 65 °C. The captured DNA:RNA hybrids were recovered using Dynabeads MyOne Streptavidin T1 (Dynal). The DNA was eluted from the beads and desalted using purified using Ampure XP beads (Agencourt).The purified capture products were then amplified using the SureSelect Post-Capture Indexing forward and Index PCR reverse primers (Agilent) for 12 cycles. Libraries were loaded onto paired end flow cells at concentrations of 4–5 pM to generate cluster densities of 600,000–800,000/mm^2^ using the Illumina cBot and HiSeq Paired end cluster kit version 3.The flow cells were sequenced to a mean depth of 80X as 101 X 2 paired end reads on an Illumina HiSeq 4,000 using TruSeq SBS sequencing kit version 3 and HiSeq data collection version 1.4.8 software. Base-calling was performed using Illumina’s RTA version 1.12.4.2. Reads from the BAM files were stripped using XYalign version 1.1.5^31^. We then mapped stripped reads to the 1,000 genomes version of GRCh38 using bwa-mem version 0.7.17^32,33^.

### Neoepitope prediction

We applied EpitopeHunter to predict neoepitopes as previously described^34^. The steps are briefly as follows: We used VarScan version 2.3.9 to call variant with the following thresholds: minimum coverage of 10, minimum variant allele frequency of 0.08, and somatic p value of 0.05^35^. Variants were annotated using variant effect predictor (VEP) version 86^36^. We generated peptides consisting of 8 amino acids (8-mers), 9 amino acids (9-mers), 10 amino acids (10-mers), and 11 amino acids (11-mers) using pvacseq version 3.0.5^37^. We used HLA-LA to perform HLA typing^38^. Binding affinity between each HLA and each peptide was calculated using the Immune Epitope Database, IEDB^39^. Individual peptides were called a potential neoepitope if their binding affinity was less than 500 nM.

## Supplementary information

Supplementary Legend

Supplementary Figure S1.

Supplementary Table S1.

Supplementary Table S2.

Supplementary Table S3.
