# Supplementary material for: Unique genomic and neoepitope landscapes across tumors: a study across time, tissues, and space within a single lynch syndrome patient
Source: Sci Rep. 2020 Jul 22;10:12190. doi: 10.1038/s41598-020-68939-7 (PMC7376229; doi:10.1038/s41598-020-68939-7)
Supplement: Supplementary file 1 — Supplementary Legend [file 41598_2020_68939_MOESM1_ESM.docx]

**Supplemental Figure 1. Recurring frameshift variant in *KMT2C*.** IGV view of ***KMT2C*^K2797fs*^**^c.8390delT^ in PTCC and TNBC biopsies.

**Supplemental Table 1. Germ line variants and predicted impact in MMR Genes.**

**Supplemental Table 2. Somatic SNVs shared across the four different cancers.**

**Supplemental Table 3. Read counts and alternate reads of shared somatic SNVs.**
