# Supplementary figures and images for: Unique genomic and neoepitope landscapes across tumors: a study across time, tissues, and space within a single lynch syndrome patient
Source: Sci Rep. 2020 Jul 22;10:12190. doi: 10.1038/s41598-020-68939-7 (PMC7376229; doi:10.1038/s41598-020-68939-7)

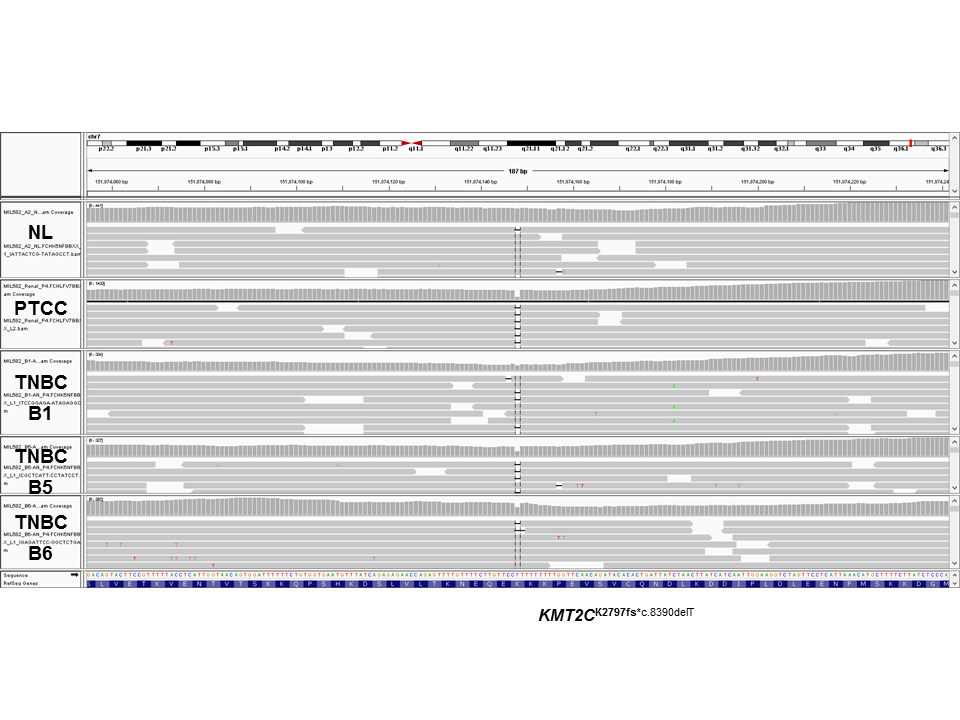

Supplement: Supplementary file 2 — Supplementary Figure S1. [file 41598_2020_68939_MOESM2_ESM.tif]

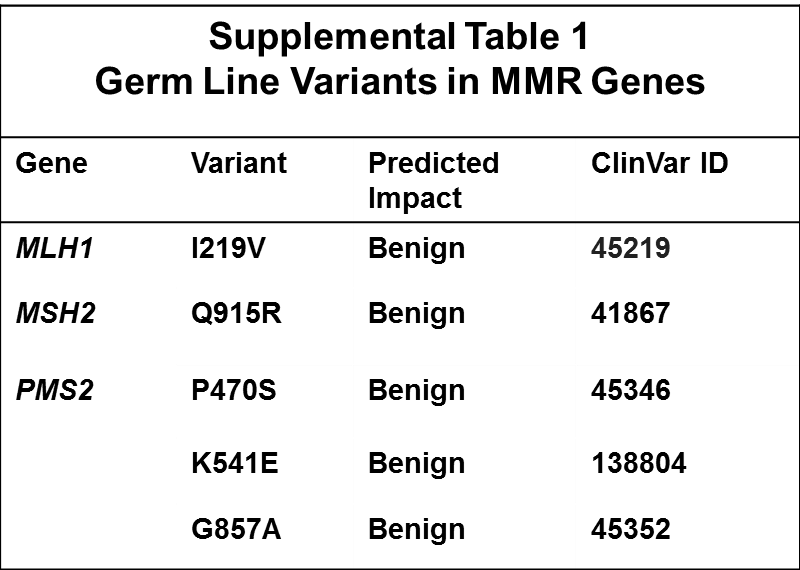

Supplement: Supplementary file 3 — Supplementary Table S1. [file 41598_2020_68939_MOESM3_ESM.tif]

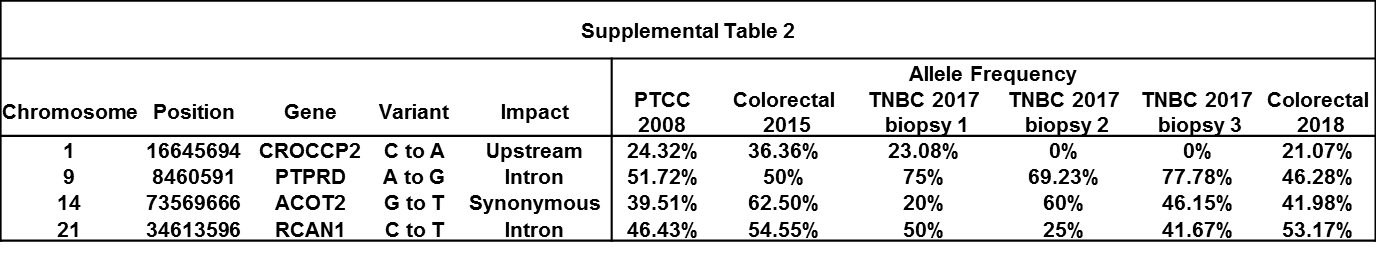

Supplement: Supplementary file 4 — Supplementary Table S2. [file 41598_2020_68939_MOESM4_ESM.tif]

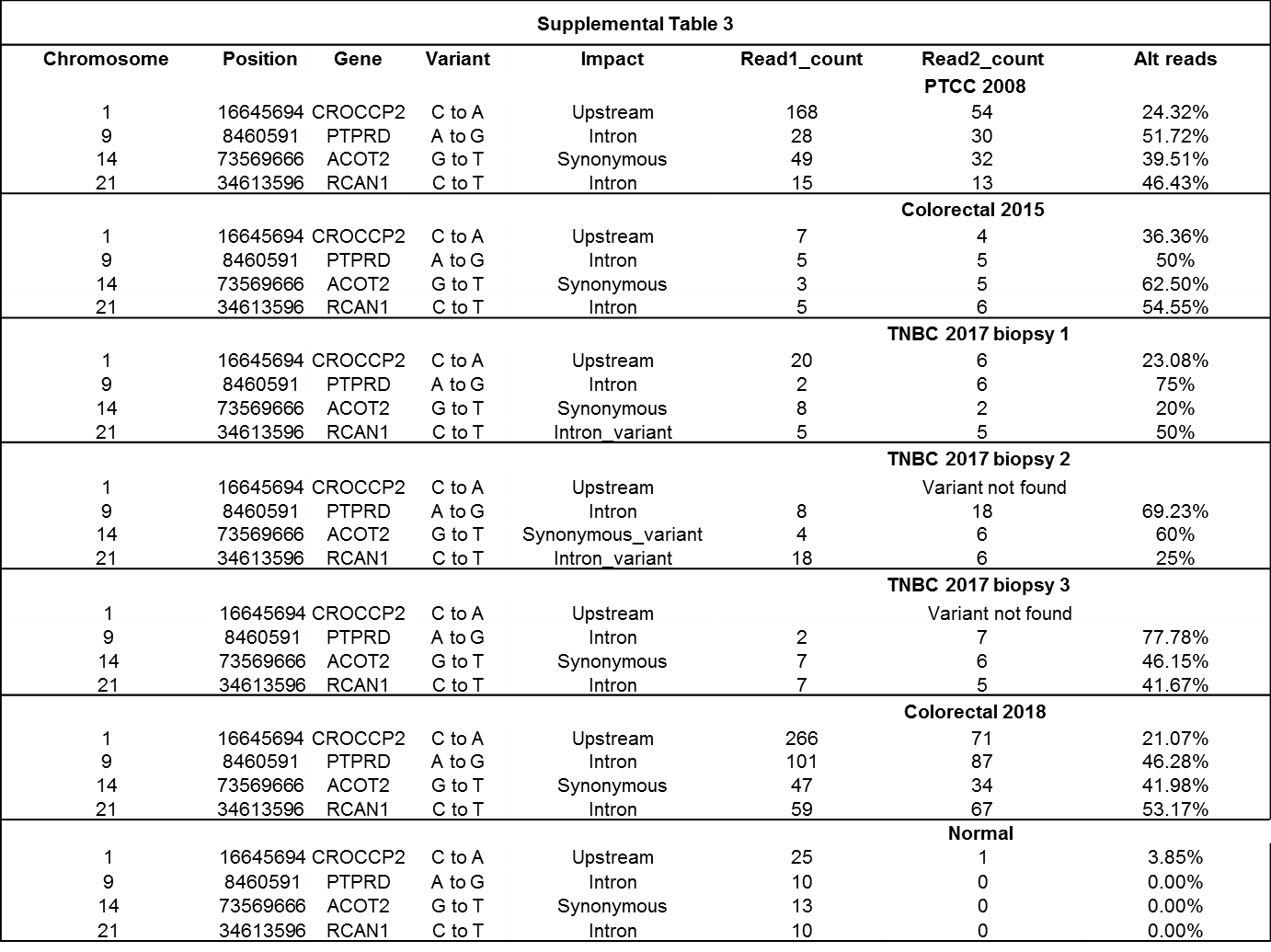

Supplement: Supplementary file 5 — Supplementary Table S3. [file 41598_2020_68939_MOESM5_ESM.tif]
